# Role of Flies as Vectors of Foodborne Pathogens in Rural Areas

**DOI:** 10.1155/2013/718780

**Published:** 2013-08-04

**Authors:** Cláudia Barreiro, Helena Albano, Joana Silva, Paula Teixeira

**Affiliations:** CBQF-Centro de Biotecnologia e Química Fina, Laboratório Associado, Escola Superior de Biotecnologia, Universidade Católica Portuguesa/Porto, Rua Dr. António Bernardino Almeida, 4200-072 Porto, Portugal

## Abstract

This study aims to evaluate flies as a vector for foodborne pathogens. For this purpose, several flies were collected from different sites from rural areas. These flies were then analyzed for the presence of Enterobacteriaceae, *Escherichia coli*, *Staphylococcus* coagulase positive, and *Listeria monocytogenes*. Another aim of this study was to evaluate some virulence factors of the collected pathogens: susceptibility to some antibiotics and the presence of enterotoxigenic *S. aureus*. The results showed that flies in the presence of animals demonstrated a significantly higher prevalence of the studied pathogens than those collected in the kitchens, and kitchens situated in the closest proximity to the animal husbandry had a higher count than the kitchens in private houses. Enterobacteriaceae was the indicator organism with the highest microbial counts followed by *E. coli* and *S. aureus*. *Listeria monocytogenes* was not detected from any of the collected flies. The antimicrobial susceptibility test showed that the bacteria carried by the flies possessed multiantibiotic resistance profiles, and enterotoxin A was produced by 17.9% of the confirmed *S. aureus* isolates. These results demonstrate that flies can transmit foodborne pathogens and their associated toxin and resistance and the areas of higher risk are those in closer proximity to animal production sites.

## 1. Introduction

Flies are “pests” of great medical and veterinary significance and are one of the most important vectors of human diseases worldwide [1, 2]. Houseflies are important nuisance pests of domestic animals and people, as well as the main fly vectors of foodborne and animal pathogens [3]. Due to their indiscriminate movements, ability to fly long distances, and attraction to both decaying organic materials and places where food is prepared and stored, houseflies greatly amplify the risk of human exposure to foodborne pathogens. Houseflies can transport microbial pathogens from reservoirs (animal manure) where they present a minimal hazard to people to places where they pose a great risk (food) [1]. Stable flies are bloodsucking insects and important pests of domestic animals and people and can cause great economic losses in the animal industry [4], and they can also play a role in ecology of various bacteria originating from animal manure and other larval developmental habitats [5]. 

Most bacteria associated with insects include foodborne pathogenic bacteria such as *Escherichia coli*, *Salmonella* spp., *Shigella *spp., and others [6]. The potential of adult houseflies to transmit pathogens such as *Campylobacter *[7, 8], *E. coli* O157:H7 [9], *Salmonella* spp. [10, 11], and *Shigella* spp. [12], between others, has been also reported. For example, it has been demonstrated that houseflies are capable of transmitting *E. coli* O157:H7 to cattle, the major reservoir of this human foodborne pathogen [13]. Fruit flies, primarily the Mediterranean fruit fly and the vinegar fruit fly, were also reported as potentially competent vectors for *E. coli* O157:H7 and were capable of contaminating fruits with this pathogen under laboratory conditions [14]. Cardozo et al. [15] identified *E. coli* and *Salmonella* spp. in a population of houseflies collected from a local milk production and manufacturing of handmade Minas cheese. Another naturally occurring association between insects and *Salmonella* spp. occurs in synanthropic flies. Because flies serve as vectors of pathogens, extensive studies have been performed that illustrate its capacity as a vehicle for transmission of pathogens [16, 17]. There is also concern that flies may also contribute to the spread of avian influenza [18]. Blunt et al. [19] also confirmed the potential of the dominant *Musca domestica* flies to act as vectors for circovirus carriage, and this potential may also be true for many nonenveloped viruses. In another perspective, Faulde and Spiesberger [20] also showed that a moth fly is a potential mechanical vector of bacterial pathogens associated with nosocomial infections.

Additionally, the development of antibiotic resistance among clinical bacterial isolates and commensal bacteria of people and animals, as well as bacteria in other habitats, raises a concern that flies may be vector competent not only for specific pathogens but also for nonpathogenic bacteria carrying antibiotic resistance genes [3]. 

This study aims to evaluate flies as vectors for foodborne pathogens. For this purpose, several flies were collected from different places, which have animals present and are near residential areas. The different collection sites were chosen to reflect the interaction of flies with different animals and the level and difference of prevalence of the studied pathogens. Therefore, the possibility of cross-contamination between these flies, with the animals, and in the nearby houses was taken into consideration during this study.

## 2. Material and Methods

### 2.1. Collection of Flies

Glue traps were placed at previously selected locations, chosen to reflect the exposure of flies to different types of animals and to different environments and places near urban areas (Table 1). Traps were exposed for approximately five hours and then collected for further analysis.

### 2.2. Preliminary Studies

In order to determine the number of flies that should be used for the further studies, different suspensions were performed: one fly in 9 mL of sterile Buffered Peptone Water (BPW, Oxoid Ltd., Basingstoke, Hampshire, UK); five flies in 9 mL of BPW; 10 flies in 9 mL of BPW; and 20 flies in 9 mL of BPW.

### 2.3. Detection and Enumeration of Pathogens

According to the results obtained in the previous section and for all subsequent analyses, five flies were added to 9 mL of BPW. This number of flies was selected, as it was possible to detect/enumerate the target organism. Appropriate decimal dilutions were prepared in Ringer's solution (LabM) until the 10^−4^ dilution for detection and microbial enumeration: enumeration and detection of Enterobacteriaceae were performed according to International Standards ISO 21528-2:2004 and ISO 21258-1:2004, respectively, [21, 22]; enumeration of *E. coli* was performed according to the ISO 16649-2 [23]; enumeration and detection of *L. monocytogenes *were performed according to ISO 11290-1 and -2, respectively, [24]; enumeration of coagulase positive *Staphylococcus* was performed according to the techniques described in Norma Portuguesa 4400-1 [25] with confirmation of colonies.

### 2.4. Antimicrobial Susceptibility Test

The determination of the minimal inhibitory concentrations (MIC) was performed by the agar dilution method and according to the methodology described in Clinical and Laboratory Standards Institute [26]. The inoculum was prepared from an overnight culture on TSA plates, by suspending some colonies in sterile Ringer's solution in order to obtain turbidity equivalent to 0.5 McFarland standards. The tested antibiotics were penicillin (Sigma Diagnostics, St. Louis, Mo., USA); ampicillin (Sigma); and tetracycline (Sigma). Assays were performed in duplicate, and two plates without antibiotics were used as control*. Staphylococcus aureus* ATCC 29213 was plated as a control. The isolates were categorized into susceptible, intermediate, and resistant according to the CLSI [26].

### 2.5. Study of the Presence of *Staphylococcus aureus* Toxins

The coagulase positive *Staphylococcus* isolates were identified to the species level according to the multiplex PCR developed by Zhang et al. [27]. *S. aureus* DSM 11729 was used as a positive control for gene *mec*A, *S. epidermidis* DSM 20044 as a negative control for gene *nuc,* and *S. aureus* ATCC 29213 as positive control for targeting 16S rRNA and *nuc* gene and negative for gene *mec*A.

The detection of staphylococcal enterotoxin genes A-E and G-J was determined according to Løvseth et al. [28]. The amplification of the target 16S rRNA gene was included as the internal control. As positive controls, different strains of *S. aureus* kindly supplied by Professor Løvseth (National Veterinary Institute, Norway) were used: 3169 for *sec*-bovine, *sed*, and *sej* genes; R5371/00 for *sea*, *seg*, *seh*, and *sei* genes; R5460/00 for *seb*, *seg*, *seh*, and *sei* genes; FRI913 for *sea*, *sec*, and *see* genes; R4774/00 as a negative control. The mixes were submitted to a program performed on a thermocycler (MyCycler, Bio-Rad, Hercules, USA) with an initial denaturation step at 94°C for 10 min, 31 amplification cycles each with 1 min at 95°C, 45 s at 62°C, and 1 min at 72°C followed by an additional extension step of 10 min at 72°C. PCR products supplemented with ethidium bromide were resolved by electrophoresis in 2% (w/v) agarose at 50 V, for 3 h, using 100–1000 bp ladder molecular size markers (Bio-Rad) as standards. DNA patterns were visualized on a UV transilluminator (Gel Documentation System 2000; Bio-Rad).

## 3. Results

The characterization of the selected sites to be studied is summarized in Table 1. A total of six study sites (one dairy, two private houses, one poultry house, one barn, and one rabbit breeding facility) and nine sites were selected to collect the flies. The results of detection and enumeration of different pathogens are shown in Table 2. The level of contamination of Enterobacteriaceae, *E. coli,* and *Staphylococcus* coagulasepositive ranges between 10^1^ and 10^3^ CFU/fly.

From the enumeration and detection of pathogens, a total of 72 isolates were taken: 33 isolates belonging to Enterobacteriaceae, 11 to *E. coli,* and 28 to *Staphylococcus* coagulasepositive. There was no detection of *L. monocytogenes*, since the enumeration was beyond the detection limit (< 1.0 × 10^1^ CFU/fly). Each isolate was characterized concerning its antimicrobial susceptibility to ampicillin, penicillin, and tetracycline. The percentage of antimicrobial susceptibility (sensitivity, resistant and intermediate) obtained for each group is presented in Table 3. The Enterobacteriaceae showed the highest resistance to penicillin (39.4%) followed by an equal percentage of resistance to both ampicillin and tetracycline. Seven isolates (21.2%) showed resistance to the three antimicrobial agents. All the *E. coli* isolates (11) were susceptible to ampicillin; 72.7% were also susceptible to penicillin; and 63.6% to tetracycline. None of the isolates showed simultaneous resistance to the three antimicrobial agents, and only two (18.2%) showed resistance to one antimicrobial agent and an intermediate value to another. All the *Staphylococcus* isolates were resistant to penicillin, 60.7% (17) were also resistant to ampicillin, and 2.8% (1) were resistant to tetracycline; 64.3% of the strains were resistant to two antimicrobial agents. None revealed resistance to all three antimicrobial agents.

From the total of 28 *Staphylococcus* coagulase-positive isolates, 42.9% (12) were confirmed as *S. aureus* (data not shown). Of the total confirmed *S. aureus*, 17.9% (5) showed the presence for the encoding gene of SEA (data not shown). According to the multiplex developed by Zhang et al. [27], the gene *mec*A was not present in any of the confirmed *S. aureus *(data not shown).

## 4. Discussion

As already stated, *Musca domestica* is a worldwide-distributed pest organism and the dominant synanthropic fly species in animal production, homes, and restaurants. The control of the housefly population is a major problem for most livestock farming, caused by the ideal breeding and feeding conditions for the flies around the farm [17]. This was one of the reasons for choosing the “rural area,” near the “farm land”, as the study sites of this work: houseflies are often found resting on animals, on their manure, and decaying food; they can pick up organisms present in those sites and transport them when they travel to a private house, to a kitchen, thus becoming high risk vectors. 

According to results presented in Table 2, the animal feeding area and the milking area showed similar and larger prevalence of the pathogens investigated. Of all the studied sites, site 1 was the one with the highest prevalence of pathogens. The collection sites “animal feeding area” and “milking area” (site 1), along with the barn (site 4, Table 2), were the only sites showing the simultaneous presence of *E. coli*, Enterobacteriaceae, and *Staphylococcus* coagulasepositive. The “workers feeding area” showed lower numbers of the organisms investigated (site 1), which reflects the fact that it is protected from the exterior, not allowing the free flow of flies from exterior to interior. In the case of the “milking area,” the importance of limiting the presence of flies is crucial, since milk must be produced, handled, and stored in a clean and sanitary environment to maintain a high quality. Public health authorities consider numerous flies in and around the dairy as evidence that sanitary standards are not being met.

Flies captured in the kitchens of private houses (sites 2 and 3, Table 2), even though in the proximity of animal production facilities and farm land, showed a significantly lower presence of pathogens and absence of Enterobacteriaceae. Comparing these kitchens, it is possible to see that the fact that one household permits the animals (dog) to walk freely in this area and the other does not, which may account for the difference in the detected organisms. Also, comparing the results of the kitchens (sites 2 and 3, Table 2) with the “workers feeding area” (site 1, Table 2), this site has a higher count of pathogens, demonstrating that even though the area in site 1 is protected from the exterior and successfully restrains a large bulk of flies from transporting bacteria to the interior, the close proximity to the presence of animals poses an increased risk of transmission of organisms. Food safety agencies have for a long time prohibited the presence of animals in restaurants, with a higher focus on the kitchen area. These results also confirmed that the presence of domestic animals (pets) in the meal preparation sites constitutes an increased risk, and the animals themselves can transmit these pathogens and contaminate the area; pathogens then can be further spread to the food and people by the flies present in the same area. Although there exists no direct connection to food safety, Urban and Broce [29] found that *M. domestica* and *C. macellaria* are the most significant transmitting vectors of pathogens in Kansas dog kennels. 

For the other study sites (sites 4, 5, and 6), it was observed that the one that poses a greater risk for the vector potential of flies, was the barn with the presence of horses (site 4, Table 2) showing a large prevalence of all the organisms studied. The poultry house (site 5, Table 2) showed the prevalence of Enterobacteriaceae including *E. coli*, and the rabbit breeding (site 6, Table 2) showed only the presence of Enterobacteriaceae. *Listeria monocytogenes* was not detected in any of the study sites. 

As we can see, there is a significantly larger prevalence of pathogens in the flies captured on the collection sites where animals were present, since houseflies commonly build up very large populations on cattle farms and other animal facilities. Although there are not so many similar studies, these results are in agreement with those described in the literature. Ahmad et al. [13] demonstrated that houseflies are capable of transmitting *E. coli* O157:H7 to the cattle digestive tract and likely play a role in the ecology of this human foodborne pathogen in the cattleproduction environment; Alam and Zurek [30] suggested that houseflies in cattle farms play a role in the dissemination of *E. coli* O157:H7 among animals and to the surrounding environment; Förster et al. [17] demonstrated the potential of the housefly as a vector of metazoan pig parasites; *Salmonella* was isolated from commercial dairies and poultry ranches, demonstrating that houseflies are potential carriers of *Salmonella* organisms and pose a possible health risk to communities living in close proximity to animal operations that harbor heavy fly population [10]. An interesting study by Holt et al. [31] indicates that flies exposed to an environment containing *Salmonella* serovar Enteritidis can become colonized with the organism and might serve as a source for its transmission within a flock situation.

Overall, only 42.9% of *S. aureus *was detected from the *Staphylococcus* coagulase-positive isolates, a number smaller than expected, because *S. aureus* is the cause of a variety of infections in both humans and animals and is a common cause of bovine, ovine, and caprine mastitis [32]. Of the total confirmed *S. aureus*, 17.9% (5) showed the presence for the encoding gene for SEA. Among the wide range of staphylococcal virulence factors, there is a growing list of secreted superantigen toxins, including enterotoxins A to O and U. Of these, SEA is the most common toxin implicated in staphylococcal food poisoning, and it is also associated with several other serious disorders. Therefore, its presence among the pathogens on the captured flies showed that they pose a high risk as vectors, especially if we consider its role in staphylococcal food poisoning and the presence of the flies in the kitchen area.

Of the studied antimicrobial agents, penicillin was the one with the lowest efficacy (59.7% resistance) for the total of the pathogens isolated from the flies, followed by ampicillin (40.3% resistance). Tetracycline revealed itself as the most effective antimicrobial agent (66.7% susceptibility) against the studied pathogens (Table 3). The increase of the number and emergence of new bacterial strains resistant to antimicrobial agents is the result of the frequent and uncontrolled uses of these agents in medicine and food animal production. When animals are treated in any way with antimicrobial agents, the pathogens that become resident in the animal and, therefore, are present in their feces are likely to be resistant to those agents. Flies live and develop in close proximity with these animals and are often present in the animal manure and have unrestricted movement. The bacterial isolates tested showed a large population with antimicrobial resistance that can be carried by the flies into the human habitat. Graham et al. [33] reported that flies collected near broiler poultry operations may be involved in the spread of drug resistant bacteria from these operations and may increase the potential for human exposure to drug-resistant bacteria. The same conclusion was reported by Davari et al. [34] in houseflies collected in hospitals and slaughterhouses. Another study reported that the majority of houseflies collected from fast-food restaurants in the USA carried a large population of antibiotic-resistant and potentially virulent enterococci, and this contamination is more frequent in summer months when house flies are more common in restaurants than in winter months [35, 36], indirectly implicating house flies as a potential source of the contamination. Our results contribute for showing the persistence of resistant bacteria in the environment and highlight the reservoir of resistance associated with the use of antibiotics as a feed additive animal industry. Further, the carriage of antibiotic resistant bacteria by flies in environment increases the potential for human exposure to drug-resistant bacteria. 

## 5. Conclusions

In this work, we showed that the hypothesis of transmission of foodborne pathogens and potential disease, as well as some virulence factors, by flies is higher in flies that have their habitat in close proximity to animal production and that the presence of domestic animals in the kitchen area in private households also poses a higher risk. The importance of limiting fly breeding by employing proper sanitation is of crucial importance. It is clear that without the use of proper sanitation methods, flies will continue to replicate and disperse from adjacent areas and will undermine any control measures. Good sanitation will result in a reduction in fly population. 

## Figures and Tables

**Table 1:** Description of the study sites used during this work.

	Study site 1	Study site 2	Study site 3	Study site 4	Study site 5	Study site 6
Area	Rural	Rural	Rural	Rural	Rural	Rural
Type	Dairy	Private house	Private house	Barn	Poultry house	Rabbit breeding
Collection sites	Animal feeding area	Kitchen	Kitchen	Animal feeding area	Animal feeding area	Animal feeding area
	Milking area workers		Dog area (near private house)			
	Workers feeding area					
Number of animals	More than 50 calves and adult cows	—	—		21 (18 chickens, 3 roosters)	15 adult rabbits
In the proximity of less than 50 meters	Farm land	Farm land	Farm land	Farm land	Farm land	Farm land
			Dairy	Poultry house	Barn	Barn
				Rabbit breeding	Rabbit breeding	Poultry house
Presence of other animals on site	No	Dogs	Dogs	—	—	—

**Table 2:** Results obtained for the detection and enumeration of different pathogens in study sites.

	*E. coli* (CFU/fly)	Enterobacteriaceae (CFU/fly)	*Staphylococcus* coagulase positive (CFU/fly)
Study site 1			
Animal feeding area	6.0 × 10^3^	1.3 × 10^4^	2.0 × 10^3^
	1.8 × 10^4^	4.0 × 10^3^	3.0 × 10^2^
	1.1 × 10^4^	EN: 1.3 × 10^1^	2.2 × 10^2^
		<1.0 × 10^1^	
Milking area	2.0 × 10^3^	1.5 × 10^3^	7.2 × 10^2^
	EN: 1.4 × 10^1^	3.2 × 10^4^	1.0 × 10^2^
	<1.0 × 10^1^		
	>3.0 × 10^6^	4.2 × 10^3^	4.8 × 10^1^
	>3.0 × 10^6^		
Workers feeding area	<1.0 × 10^1^	7.0 × 10^1^	0.5 × 10^1^
Study site 2			
Kitchen	<1.0 × 10^1^	EN: 1.1 × 10^1^	<1.0 × 10^1^
	<1.0 × 10^1^	<1.0 × 10^1^	
	<1.0 × 10^1^	<1.0 × 10^1^	<1.0 × 10^1^
	<1.0 × 10^1^	<1.0 × 10^1^	<1.0 × 10^1^
Study site 3			
Kitchen	<1.0 × 10^1^	3.8 × 10^1^	EN: 1.3 × 10^1^
			<1.0×10^1^
Dog area	<1.0 × 10^1^	<1.0 × 10^1^	3.8 × 10^2^
Study site 4			
Barn	1.6 × 10^3^	1.5 × 10^3^	1.5 × 10^3^
Study site 5			
Poultry house	3.6 × 10^2^	8.8 × 10^1^	<1.0 × 10^1^
Study site 6			
Rabbit breeding	<1.0 × 10^1^	1.7 × 10^3^	<1.0 × 10^1^

EN: estimated number. Each sample was constituted by 5 flies. Results are presented in CFU/fly. *L. monocytogenes *was beyond the detection limit (<1.0 × 10^1^ CFU/fly) in all samples.

**Table 3:** Results of the antimicrobial susceptibility for the tested microorganisms.

	Ampicillin			Penicillin			Tetracycline		
	S	I	R	S	I	R	S	I	R
*E. coli *	11 (100%)	0 (0%)	0 (0%)	8 (72.7%)	1 (9.1%)	2 (18.2%)	7 (63.6%)	2 (18.2%)	2 (18.2%)
*Enterobacteriaceae *	21 (63.6%)	0 (0%)	12 (36.4%)	12 (36.4%)	8 (24.2%)	13 (39.4%)	15 (45.4%)	6 (18.2%)	12 (36.4%)
*Staphylococcus* coagulase positive	11 (39.3%)	0 (0%)	17 (60.7%)	0 (0%)	0 (0%)	28 (100%)	26 (94.4%)	1 (2.8%)	1 (2.8%)
*S*. aureus*	4 (33.3%)	0 (0%)	8 (66.7%)	0 (0%)	0 (0%)	12 (100%)	11 (91.7%)	0 (0%)	1 (8.3%)

*Within the coagulase-positive *Staphylococcus* results. S: susceptible, I: intermediate, and R: resistant.
